# Effects of Physical Exercise on MuRF-1/*TRIM63 mRNA* Expression in Humans: A Systematic Review

**DOI:** 10.3390/genes16020153

**Published:** 2025-01-26

**Authors:** Leonardo Henrique Silva Fagundes, Eduardo Mendonça Pimenta, Varley Teoldo da Costa

**Affiliations:** Sport Psychology Laboratory, UFMG Soccer Science Center, Department of Sport Sciences, Universidade Federal de Minas Gerais (UFMG), Belo Horizonte 31270-901, Brazil; empimenta@uol.com.br (E.M.P.); vtcosta@hotmail.com (V.T.d.C.)

**Keywords:** MuRF-1/*TRIM63*, muscle protein degradation, physical performance, gene expression, human skeletal muscle signaling

## Abstract

Background/Objectives: Muscle-specific RING finger protein 1 (MuRF-1) is a pivotal regulator of muscle protein breakdown, an essential process for post-exercise muscle adaptation. This systematic review aimed to evaluate the effects of physical exercise on MuRF-1 *mRNA* expression in humans. Methods: A literature search was conducted in PubMed, Scopus, Cochrane Library, Google Scholar, and Web of Science following the PRISMA guidelines. The search was limited to studies published from 1 January 2001 to 1 December 2024. The inclusion and exclusion criteria were defined using the PICOS strategy. Two investigators independently performed the study selection, data extraction, and assessment of methodological quality, with any disagreements resolved by a third investigator. The PEDro scale was used to evaluate the risk of bias. Results: Forty-six studies met the eligibility criteria and were included. The findings evidenced that physical exercise significantly modulates MuRF-1 *mRNA* expression in humans. Resistance exercise induces transient increases, typically peaking between 1 and 4 h, whereas endurance exercise elicits similar responses within 40 min to 4 h post-exercise. Combined exercise protocols that include resistance and endurance exercises significantly increased MuRF-1 *mRNA* expression at 3 h post-exercise. The effects of physical exercise on MuRF-1 *mRNA* expression are influenced by factors such as exercise order, intensity, contraction mode, age, sex, and fitness level. Conclusions: This systematic review shows that MuRF-1 *mRNA* expression is significantly modulated by physical exercise in humans and is sensitive to different exercise modalities. These findings suggest that this key protein involved in muscle protein breakdown and turnover is essential for exercise-induced adaptations, contributing to skeletal muscle recovery and remodeling after exercise.

## 1. Introduction

Muscle-specific RING finger protein 1 (MuRF-1), encoded by the human tripartite motif containing 63 (*TRIM63* rs2275950) gene, was initially identified by Centner et al. [1] as a myofibrillar protein with a potential role in regulating the kinase domain of titin, a large sarcomeric protein [2]. Subsequently, MuRF-1 was investigated by a group of researchers who identified its E3-ubiquitin ligase activity, suggesting the involvement of this protein in skeletal muscle atrophy [3]. Since then, studies have been performed to elucidate this specific gene’s function, signaling pathways, and regulatory mechanisms, which are not restricted to atrophy processes but may also be important for muscle protein turnover and exercise-induced adaptation [4,5,6]. Studies have demonstrated that MuRF-1 plays a vital role in muscle protein breakdown through the ubiquitin–proteasome system (UPS), a critical process for post-exercise muscle adaptation [6,7]. The UPS is a vital proteolytic pathway involved in catabolic processes (e.g., skeletal muscle atrophy) that are characteristic of various diseases and the negative consequences of treatments and life prognoses in patients [8,9].

In healthy individuals, muscle mass and, consequently, muscle protein turnover are a continuous cellular process regulated by the balance between muscle protein synthesis (MPS) and muscle protein breakdown (MPB) [10,11]. After exercise, there is a rapid and transient increase in MPS [12]. However, MPB also increases after exercise, with a shorter duration than MPS [10,11,12,13]. In this process, MuRF-1 *mRNA* expression may increase UPS activity and influence adaptive outcomes of the transcriptome to regulate physiological demands [6]. Physical exercise modulates the gene expression of proteins involved in the synthesis and degradation pathways, contributing to muscle adaptation processes [10]. MuRF-1 plays a pivotal role in muscle protein turnover and net protein balance, which are critical for skeletal muscle adaptation to acute and chronic exercise [11,12]. Exercise-induced extracellular stress signals trigger transient changes in intramuscular signaling, leading to gene transcription and protein translation alterations. These processes facilitate muscle repair and remodeling, particularly during recovery periods between exercise sessions [6,14]. Muscle remodeling occurs in response to the demands imposed by exercise and is mediated by an individual’s genetic profile [15]. Thus, gathering information about MuRF-1’s involvement in the proteolysis process will provide further insight into the molecular mechanisms underlying muscle responses and adaptation to physical exercise. This allows for a deeper examination of the influence of acute and chronic training variables and how manipulations affect intramuscular adaptations and MuRF-1 *mRNA* expression.

Most of the available studies describe the various pathological and physiological conditions to which MuRF-1 has been linked. While some studies focus on health conditions, such as skeletal muscle atrophy [3,16], cardiomyopathies [17], and immune-mediated necrotizing myopathy [18], other investigations specifically examine the relationship between MuRF-1 and exercise-induced muscle damage [19] or muscle injury occurrence in athletes [20]. A previous literature review summarized the data on MuRF-1 obtained over the last 20 years [4]. The findings highlighted its various identified functions, structure, localization, and the mechanisms of regulation and signaling. Regarding systematic reviews, one study focused on various pathological models that altered MuRF-1 gene expression in mice [21]. Another review discussed the impact of training combined with whey protein supplementation on MuRF-1 *mRNA* expression in murine models [22]. The focus of the systematic reviews mentioned was animal studies, which may result in a limited understanding, as these studies evaluated the response of MuRF-1 under physiological conditions and pathological states. Pathologies impair physiological processes and can alter an organism’s functional, structural, and biochemical responses [4,18].

It is, therefore, important to elucidate the effects of physical exercise on MuRF-1 *mRNA* expression in healthy individuals to understand the exercise response at the molecular level and its influence on skeletal muscle. Previous studies have not sufficiently addressed the responses of MuRF-1 to different exercise regimens in healthy populations, particularly in relation to distinct exercise modalities. Resistance exercise induces transient spikes in MuRF-1 expression associated with acute muscle repair and remodeling [10,12], while endurance exercise promotes sustained responses linked to metabolic adaptations and protein turnover [11]. Combined protocols result in interactions influenced by exercise sequence and intensity [11,12]. These results demonstrate the regulatory effects of different exercise modalities on MuRF-1 expression, emphasizing its role in muscle mass regulation and adaptation in humans. Therefore, the merit of this systematic review was focused on experimental studies that describe the molecular mechanisms influencing muscle protein breakdown and turnover processes in response to the effects of exercise in different populations. This review sought to provide insights that support the maintenance of skeletal muscle integrity during the recovery process following physical exercise in humans. To date, no systematic reviews have been identified that evaluated studies investigating the specific effects of physical exercise on the regulation and expression of MuRF-1 in healthy humans.

The evidence on the effects of acute and chronic physical exercise is limited, representing a prerequisite to understand the consequences of changes in MuRF-1, such as intracellular signaling, gene transcription, and protein translation in humans. Therefore, a systematic review of studies that used different physical exercise protocols to assess their isolated responses and subsequent post-exercise time course effects on MuRF-1 is needed to advance the current state of evidence on this topic. This systematic review aimed to evaluate the effects of physical exercise on MuRF-1 *mRNA* expression in humans.

## 2. Materials and Methods

### 2.1. Study Design

This systematic review followed the Cochrane guidelines [23] and was conducted according to the Preferred Reporting Items for Systematic Reviews and Meta-Analyses (PRISMA) statement [24,25] (Supplementary File S1: PRISMA checklist). The study protocol was registered in the PROSPERO database (CRD42024611778).

### 2.2. Eligibility Criteria

A comprehensive search of the literature conducted in PubMed, Scopus, Cochrane Library, Google Scholar, and Web of Science was performed from 1 January 2001 to 1 December 2024. The search strategy included Medical Subject Heading (MeSH) terms, free words, and Boolean operators. The search terms included the following: (muscle ring finger protein 1 OR MuRF-1 OR *TRIM63* OR atrogenes OR proteolytic gene expression) AND (physical exercise OR endurance training OR endurance exercise OR resistance training OR resistance exercise) AND (human skeletal muscle OR humans OR men OR women). The search strategies for each database can be viewed in Supplementary Table S2. In addition, references cited in the retrieved studies were also screened manually to identify additional eligible articles. Two independent investigators (L.H.S.F. and E.M.P.) reviewed titles and abstracts and verified potential full texts. Studies were included if they fulfilled our eligibility criteria. Disagreements between investigators were resolved by a third investigator (V.T.d.C.).

### 2.3. Inclusion and Exclusion Criteria

This systematic review was conducted according to the Population, Intervention, Comparison, Outcomes, and Study (PICOS) design strategy [26]:

1. Population: Studies included human participants aged 18 years or older who were classified as healthy, regardless of whether they were trained or untrained.
2. Intervention: Physical exercise protocols, including resistance training, endurance training, or a combination of both.
3. Comparator: Comparisons focused on the effects of resistance training, endurance training, or combined protocols on MuRF-1 *mRNA* expression, stratified by participants’ fitness levels.
4. Outcome: The primary outcome was the effects of physical exercise on MuRF-1 *mRNA* expression in human skeletal muscle.
5. Study design: Eligible studies included randomized controlled trials (RCTs), longitudinal, within-subject, crossover, and cross-sectional designs. Eligible articles needed to be written in English.

The following studies were excluded: (I) studies involving participants with any diagnosed medical condition (e.g., cancer) or musculoskeletal disorder limiting their physical performance, (II) studies implementing rehabilitation protocols, (III) studies involving blood-flow-restricted exercise, (IV) studies using hormonal and drug treatments, (V) in vitro or in vivo studies assessing animal models, (VI) studies including supplementation (e.g., whey protein), and (VII) studies without an experimental design, such as clinical reports, books, reviews, editorial letters, and conference abstracts.

### 2.4. Data Extraction

Two investigators (L.H.S.F. and E.M.P.) extracted the individual characteristics and outcome data from the included trials. Disagreements between investigators were settled by a third investigator (V.T.d.C.). A custom spreadsheet for data analysis was created with Microsoft Excel. The extracted data included the authors, aim investigated, participant characteristics, biopsy time points, exercise protocol, and results related to the main effects of MuRF-1. A qualitative approach was employed to synthesize the results due to the heterogeneity of the included studies regarding their design, participant characteristics, exercise protocols, and outcome measurement methods. The main findings were categorized based on exercise type (resistance, endurance, or both) and the impact on distinct subgroups, such as trained, physically active, and untrained individuals. A quantitative analysis was not performed due to the lack of standardized data required for a robust meta-analysis.

### 2.5. Quality Assessment

The Physiotherapy Evidence Database (PEDro) scale was used to evaluate the methodological quality of the included studies [27]. Two independent investigators (L.H.S.F. and E.M.P.) assessed the score for each study using the PEDro scale, and discrepancies were addressed by a third investigator (V.T.d.C.). No automation tools were utilized during this process.

### 2.6. Study Selection

**Figure 1:**
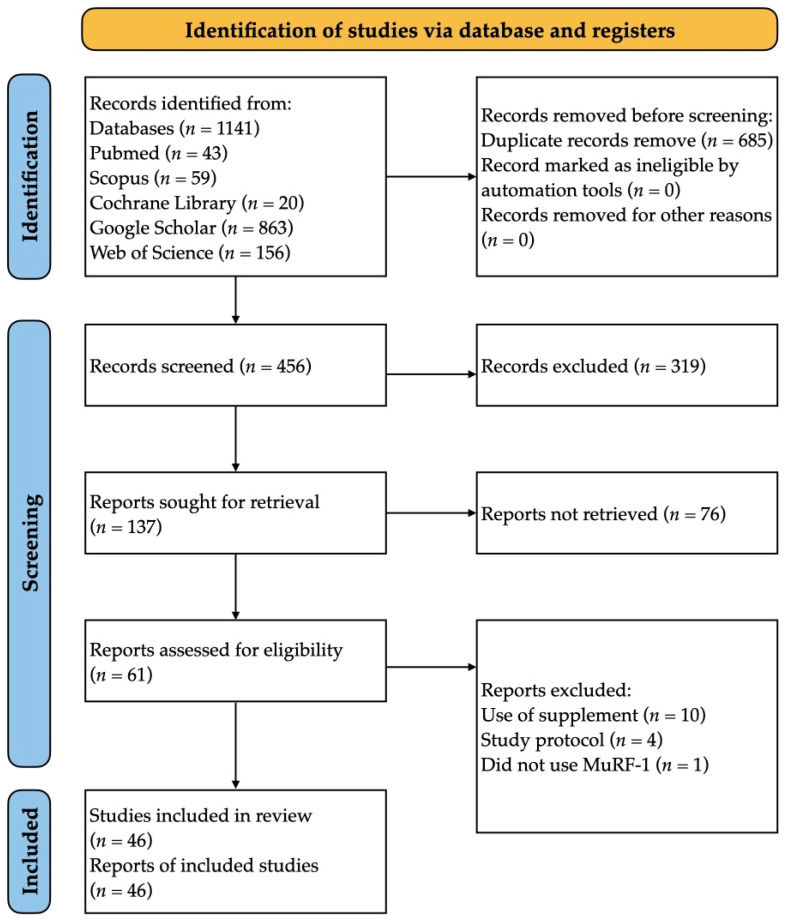
PRISMA flow diagram of study selection.

A total of 1141 records were identified through database searches. After removing 685 duplicates, 137 titles and abstracts were screened. Subsequently, 61 full texts were evaluated for eligibility, leading to the inclusion of 46 studies in the final analysis [28,29,30,31,32,33,34,35,36,37,38,39,40,41,42,43,44,45,46,47,48,49,50,51,52,53,54,55,56,57,58,59,60,61,62,63,64,65,66,67,68,69,70,71,72,73]. A diagram of the flow of study selection is provided in Figure 1.

## 3. Results

### 3.1. Study Characteristics

The selected studies, published between 2005 and 2023, were classified into those with a within-subject [30,35,40,47,48,49,51,52,53,54,55,62,64,73], longitudinal [38,41,42,44,45,46,50,57,60,63,66,68,69,72], cross-sectional [29,33,34,36,56,58,65,67,71], randomized clinical trial [31,32,37,59,70], and crossover design [28,39,43,61]. The physical exercises described in the studies included resistance exercise [29,30,32,33,34,36,38,43,44,47,48,55,56,58,65,66,67,70,71,72,73], endurance exercise [40,41,42,45,46,50,60,61,62,63,69], and combined resistance and endurance protocols [28,31,35,37,39,49,51,52,53,54,57,59,64,68]. Participants were categorized as trained [28,30,32,40,42,45,50,54,58,59,60,62,70], physically active [31,34,35,36,37,39,41,44,47,48,49,51,52,53,63,67,69,71], and untrained [29,33,34,38,43,46,55,56,57,58,61,64,65,66,68,72,73]. The studies involved men only [28,30,31,32,33,35,37,39,40,42,43,44,45,47,48,50,51,52,53,55,57,58,59,60,61,62,63,64,67,68,69,70,73], women only [34,38,46,65,72], or both men and women [29,36,41,49,56,66,71]. The sample sizes varied from 6 to 87 participants, with ages ranging from 20.0 to 85.2 years. Table 1 provides an overview of the study details.

### 3.2. Study Quality Assessment

The PEDro scores of the included studies ranged from 3 to 7 points out of 10 (average = 4 points). Fifteen studies (38%) showed moderate methodological quality (i.e., scores ≥ 5 points), while three studies (8%) were classified as having a low risk of bias (i.e., scores ≥ 6 points). The main reasons for increasing the risk of bias were not blinding therapists (46 studies, 100%), not blinding participants (46 studies, 100%), and not blinding assessors (39 studies, 85%). The evaluation of the included studies did not reveal direct evidence of missing results. The certainty of evidence for the outcomes assessed was moderate, primarily due to limitations related to the risk of bias and heterogeneity among the included studies. A detailed risk assessment of bias is presented in Table 2.

### 3.3. Effects of Resistance Exercise on MuRF-1 mRNA Expression

Four studies investigated the link between resistance exercises and trained individuals [30,32,58,70]. Churchley et al. [30] evaluated whether pre-exercise muscle glycogen content influences gene transcription. The researchers found that MuRF-1 *mRNA* expression was higher in the control leg (Norm leg) compared with the Low leg (three-fold, ES = 0.6, *p* < 0.05). Coffey et al. [32] examined acute molecular responses through repeated sprint (SPR) and resistance exercise (RE), in which they verified a significant increase in MuRF-1 *mRNA* expression that was elevated above rest following 3 h of recovery from RE1-SPR2 and SPR1-RE2 (ES > 0.1, *p* < 0.01). It is noted that the MuRF-1 *mRNA* abundance was moderately exacerbated when SPR was undertaken after RE (RE1-SPR2 vs. SPR1-RE2, ~25%, ES = 0.75). Mikkelsen et al. [58] displayed a significant age × training interaction (*p* = 0.022) and verified that MuRF-1 *mRNA* expression was lower in trained individuals (*p* < 0.001). Furthermore, Vann et al. [70] showed after 6 weeks of training that MuRF-1 *mRNA* expression did not exhibit a group × time interaction (*p* = 0.567) or a main effect of the group (*p* = 0.463). MuRF-1 *mRNA* expression did, however, exhibit a main effect of time (*p* < 0.001; CI_PRE_ = 1.00–1.00; CI_POST_ = 4.46–6.37; CI_DL_ = 6.44–11.06), where MuRF-1 *mRNA* expression was greater at DL than at pre- and post-exercise (*p* < 0.001). Moreover, MuRF-1 *mRNA* expression was greater at post- than at pre-exercise (*p* < 0.001).

Six studies assessed the effects of resistance exercise on physically active individuals [34,36,44,47,48,67]. Drummond et al. [34] observed a significant reduction in MuRF-1 *mRNA* expression in inactive and frail older women compared with their active healthy counterparts (*p* = 0.01). Fry et al. [36] observed that MuRF-1 *mRNA* expression increased 3 h after resistance exercise in younger and older groups (*p* < 0.05). Moreover, MuRF-1 *mRNA* expression was also significantly elevated above rest at 6 h post-exercise in both groups (*p* < 0.05). Léger et al. [48] found that MuRF-1 *mRNA* expression after an 8-week program (post-Tr) was significantly higher than that in the period before training (pre-Tr) and 8 weeks following the last training session (post-De-Tr) in physically active men (*p* < 0.01). Koskinen et al. [47] identified a positive and significant correlation between MuRF-1 *mRNA* expression and immediate post-exercise levels (r = 0.73, *p* = 0.039), while MuRF-1 *mRNA* levels demonstrated a negative correlation with jump height 3 h post-exercise (r = −0.75, *p* = 0.019). Stefanetti et al. [67] detected significant upregulation in both younger (1–5-fold) and older (1.3-fold) subjects 2 h post-exercise (*p* < 0.05) and a significant effect of exercise (*p* < 0.01). However, Kern et al. [44] reported that isokinetic (ISO-K) and vibrational–proprioceptive (VIB) protocols did not influence MuRF-1 *mRNA* expression pre or post training.

Eleven studies measured the effects of resistance exercise on untrained individuals [29,33,38,43,55,56,65,66,71,72,73]. Yang et al. [73] verified changes in MuRF-1 *mRNA* expression in slow-twitch (MHC I) and fast-twitch (MHC IIa) fibers following resistance exercise. MuRF-1 *mRNA* levels increased at 4 h after resistance exercise in both MHC I (2.2-fold) and MHC IIa fibers (4.8-fold) (*p* < 0.05), but they returned to the basal levels by 24 h post-exercise in both fiber types. Repeated resistance exercise induced an increase in MuRF-1 *mRNA* expression 2 h post-exercise in both exercise sessions (*p* < 0.05); this increase was 2.0-fold after the first session and 1.6-fold after the second session [55]. Skelly et al. [66] verified a significant increase in MuRF-1 *mRNA* expression at 3 h compared with the baseline and immediately post-exercise, with similar responses in men and women (*p* < 0.001). Baumert et al. [29], evaluating the response to exercise-induced muscle damage, reported a significant increase in MuRF-1 *mRNA* expression following acute resistance exercise (*p* < 0.05). However, Kamandulis et al. [43] analyzed MuRF-1 *mRNA* expression using drop jumps and found no significant differences over time or between the two protocols (DJ-5 min and DJ-20 s). Dalbo et al. [33] observed that baseline MuRF-1 *mRNA* expression was higher in older men compared to younger men (*p* < 0.05). Merritt et al. [56] investigated both sexes and divided them into three groups (AGE40, AGE61, and AGE76). After subjects performed unaccustomed resistance exercises, it was shown that MuRF-1 *mRNA* expression was higher in AGE76 than in AGE40 at baseline (*p* < 0.05), and both age groups experienced a decrease in MuRF-1 to similar levels 24 h after unaccustomed resistance exercises (*p* < 0.05). On this path, but investigating only women, Raue et al. [65] compared two groups (old vs. young women), and at rest, OW expressed higher MuRF-1 *mRNA* expression than YW (*p* = 0.04). In response to resistance exercise, there was an age effect, in which both YW and OW had an induction in MuRF-1 (YW: 3.6-fold, 95% CI = 2.8–4.4; OW: 2.6-fold, 95% CI = 1.9–3.2, *p* = 0.001). Williamson et al. [72] investigated women (YW and OW) of a similar age and revealed after 12 weeks of progressive resistance training that YW displayed a downregulation of MuRF-1 (−29%, *p* < 0.05). After training, OW showed significantly higher MuRF-1 *mRNA* expression than YW (*p* < 0.05). In contrast, YW and OW completed 12 weeks of resistance exercise training and did not show significant differences in MuRF-1 *mRNA* expression [38]. Moreover, Whitman et al. [71] revealed that MuRF-1 *mRNA* expression was not different between subjects who were old and young or between men and women.

### 3.4. Effects of Endurance Exercise on MuRF-1 mRNA Expression

Six studies examined the effects of endurance exercise on trained individuals [40,42,45,50,60,62]. Harber et al. [40] evaluated the metabolic response of the vastus lateralis and soleus muscles after running and found that MuRF-1 *mRNA* expression was upregulated at 4 h post-exercise in the vastus lateralis only (*p* < 0.05). Pasiakos et al. [62] detected significantly increased MuRF-1 *mRNA* expression (4.7-fold, immediately post-exercise; 5.7-fold, 3 h post-exercise) compared with the rest of the time points (*p* < 0.001). Jamart et al. [42] showed that MuRF-1 *mRNA* expression increased immediately at the end of ultra-endurance running compared with 2 h before exercise (71 ± 31%, *p* = 0.023). Similarly, Kim et al. [45] reported a significant 583.0 ± 244.3% (*p* = 0.024) increase in MuRF-1 *mRNA* expression 3 h following ultra-endurance exercise. Luden et al. [50] demonstrated a significant increase in MuRF-1 *mRNA* expression (*p* < 0.05) after exercise before and after a taper, with a reduced response being observed post-taper (2.3-fold vs. 1.7-fold, *p* < 0.05). In contrast, Murach et al. [60] observed no changes in MuRF-1 *mRNA* expression during exercise in the heavily trained and tapered states.

Three studies verified the effects of endurance exercise on physically active individuals [41,63,69]. Popov et al. [63] investigated the effects of a 2-month aerobic training program and observed a significant reduction in MuRF-1 *mRNA* expression in the endurance-trained state compared with the untrained state (*p* < 0.01). In this line, the twelve-day cycling protocol was able to attenuate MuRF-1 *mRNA* expression compared with day 1 in women and men [41]. Meanwhile, Valladares-Ide et al. [69] reported no changes in MuRF-1 *mRNA* expression after any cycling exercise.

In untrained individuals, Nedergaard et al. [61] assessed men who performed step exercises using eccentric work with one leg and concentric work with the other leg. MuRF-1 *mRNA* expression showed strong upregulation with concentric loading compared with pre-exercise (*p* < 0.01) and eccentric loading (*p* < 0.001). Konopka et al. [46] demonstrated that 12 weeks of aerobic exercise training on a cycle ergometer could not alter MuRF-1 *mRNA* expression in older women.

### 3.5. Effects of Resistance and Endurance Exercise on MuRF-1 mRNA Expression

Three studies evaluated MuRF-1 *mRNA* expression in response to resistance and endurance exercise in trained individuals [28,54,59]. Moberg et al. [59] found that MuRF-1 *mRNA* expression at 90 min and 180 min (RE-Arm) was higher than the baseline (*p* < 0.05), and at 90 min, ER-Arm was higher than R-Arm (*p* < 0.05). Apró et al. [28] verified that MuRF-1 *mRNA* expression increased 2.2- and 1.6-fold (*p* < 0.05) at 90 and 180 min after interval cycling followed by resistance exercise in an ER trial compared with an R trial and pre-exercise (*p* < 0.05). Lysenko et al. [54] demonstrated that MuRF-1 *mRNA* expression was only increased after aerobic exercise (40 min, 2.4-fold, *p* = 0.05) and remained the same after a combined load.

In physically active individuals, four studies used the same training protocol, which consisted of one leg performing endurance and resistance exercise (AE + RE), while the opposite limb was subjected to resistance exercise (RE) only [35,51,52,53]. Lundberg et al. [51] examined the impact of an acute aerobic exercise session on molecular adaptations to subsequent resistance training. MuRF-1 *mRNA* levels were comparable between AE + RE and RE (interaction, *p* = 0.077), showing a slight decline over time (main effect: F = 4.0, *p* = 0.038). In this line, Lundberg et al. [52] evaluated the muscle hypertrophy response after men performed a 5-week training protocol. MuRF-1 *mRNA* expression showed no significant differences when analyzing the RE leg before (pre) and both legs (AE + RE and RE) 72 h after the last training session (*p* > 0.05). Lundberg et al. [53] noted that MuRF-1 *mRNA* expression increased 3 h after (post) AE + RE compared with the opposite leg (*p* < 0.05) and the same leg at pre-exercise (2.9-fold, *p* = 0.003). Fernandez-Gonzalo et al. [35] compared the acute muscular response 3 h post-exercise using the same sample (*n* = 10 men, 25.0 ± 4.0 years) and training protocol as those in the previous study. The levels of MuRF-1 *mRNA* expression were higher in AE+ RE than in RE in the pre-exercise period (2.2-fold, *p* < 0.005). There was a time × MuRF-1 *mRNA* expression interaction in the untrained state before 5 weeks of training (F = 11.8, *p* = 0.007). In the trained state, there was a time × leg interaction (F = 33.3, *p* < 0.005), and MuRF-1 *mRNA* expression decreased from pre- to post-exercise in AE + RE, with no change in RE (1.5-fold, *p* = 0.003). MuRF-1 *mRNA* expression were greater following RE than AE + RE at 3 h post-exercise (2.0-fold, *p* < 0.005). A condition × leg interaction (F = 36.4, *p* < 0.005) highlighted a reduction in MuRF-1 *mRNA* with training in AE + RE (*p* < 0.005), while levels remained stable in RE. Additionally, in the untrained state, MuRF-1 *mRNA* was elevated in AE + RE compared to RE (*p* = 0.002). Conversely, the trained state revealed the opposite trend, with MuRF-1 *mRNA* being lower in AE + RE relative to RE (*p* = 0.028).

Coffey et al. [31] examined the acute molecular response to divergent exercise stimuli by combining consecutive bouts of resistance (RE) and endurance exercise (EE) in physically active individuals. MuRF-1 *mRNA* expression was elevated from resting values when RE preceded EE (*p* = 0.009). MuRF-1 transcriptional activity was exacerbated when EE was undertaken after RE (RE-EE vs. EE-RE, ~52%, ES = 0.4). Louis et al. [49] measured the time course of MuRF-1 *mRNA* expression in physically active men and women after an acute bout of resistance (RE) and running (RUN) exercise. Following RE, MuRF-1 *mRNA* expression increased 3.5-fold at 1 h, 3.4-fold at 2 h, and 2.0-fold at 4 h post-exercise compared with the pre-exercise level (*p* < 0.05). After RUN, MuRF-1 *mRNA* expression increased 2.7-fold at 1 h, 3.6-fold at 2 h, and 1.8-fold at 4 h post-exercise (*p* < 0.05). Hansson et al. [39] evaluated the responses to resistance exercise of the elbow extensors and found that there was an interaction effect for MuRF-1 *mRNA* (*p* = 0.003) due to greater expression in AE + RE from pre- to post-exercise (3.9-fold, *p* = 0.001) compared with RE. In addition, there were differences in the MuRF-1 *mRNA* expression in AE + RE for the opposite arm within the same time points of post1 and post2 (*p* < 0.05). Fyfe et al. [37] compared the effects of concurrent training on physically active men who performed RE only, HIIT + RE, and MICT + RE, and they identified a small increase in MuRF-1 *mRNA* expression at 3 h for both MICT ± RE (535 ± 464%; ES = 0.33 ± 0.20; *p* = 0.016) and HIT ± RE (585 ± 684%; ES = 0.52 ± 0.64; *p* = 0.170) compared with RE. However, MuRF-1 *mRNA* expression was not altered by RE at 3 h compared with REST, and there were no differences in MuRF-1 *mRNA* expression between HIT + RE and MICT + RE at any time point.

In untrained men, Stefanetti et al. [68] examined molecular responses in subjects who underwent 10 weeks of endurance (ET) or resistance training (RT), followed by a single session of either endurance exercise (EE) or resistance exercise (RE). A significant group × time interaction showed that ET increased MuRF-1 *mRNA* expression by 138 ± 24% (*p* < 0.01). EE increased MuRF-1 *mRNA* at 2.5 h (340 ± 94%, *p* < 0.001). In EE, MuRF-1 *mRNA* expression was greater at 0 h, 2.5 h, and 22 h when compared with RE (*p* < 0.001). Michel et al. [57] investigated the effects of resistance and high-intensity interval training on skeletal muscle proteolytic markers and showed that MuRF-1 *mRNA* expression demonstrated model significance (*p* = 0.002), where post- was greater than both pre-exercise (*p* = 0.004) and MID (*p* = 0.032), with no differences in pre and MID (*p* > 0.999). Pugh et al. [64] reported a 4.6-fold increase in MuRF-1 *mRNA* expression at 2 h and a 1.6-fold elevation at 6 h after RE + HIIT (*p* < 0.05), while RE alone showed no changes in expression over time.

## 4. Discussion

This systematic review evaluated the effects of physical exercise on MuRF-1 *mRNA* expression in humans to determine which type of physical exercise (resistance, endurance, or both) has the most empirical evidence to date. This is the first systematic review to explore the skeletal muscle molecular responses of the *TRIM63* gene within the context of physical exercise in humans. The main findings indicate that endurance and resistance exercises elicit a similar peak in the time course of MuRF-1 *mRNA* expression, occurring approximately 1–4 h after exercise. The combination of resistance and endurance exercise induces a significant increase in MuRF-1 *mRNA* expression at 3 h post-exercise. Additionally, exercise modalities (endurance and resistance), muscle contraction (eccentric and concentric), level of fitness (trained, physically active, untrained), sex (men and women), and muscle age (young and old) significantly influence MuRF-1 *mRNA* expression in humans. Therefore, a critical intra- and inter-study evaluation of methodological designs was also conducted to investigate the main mechanisms regulating MuRF-1 *mRNA* expression in response to physical exercise.

MuRF-1 *mRNA* transcription is regulated by different stimuli that activate signaling pathways, with the Forkhead box O (FoxO) family of transcription factors, particularly FoxO1 and FoxO3, serving as key regulators of MuRF-1 *mRNA* expression in response to anabolic and catabolic signals [4,48]. These signaling pathways are influenced by physical exercise, which modulates the release of hormones that coordinate metabolic and cellular activities throughout the human body [32,33,62]. Under anabolic stimulation, muscle protein synthesis is promoted through the activation of the phosphatidylinositol 3-kinase PI3K-Akt pathway, which suppresses FoxO transcription factors, thereby preventing the upregulation of MuRF-1 *mRNA* and reducing proteolytic activity [4,72].

MuRF-1 plays an important role in muscle protein breakdown, a process essential for skeletal muscle adaptation to acute and chronic exercise [3,5,9]. Protein degradation is critical for maintaining cellular homeostasis and muscle protein quality [7,8,14]. During this process, the time course of MuRF-1 *mRNA* expression varies between exercise modalities. Following resistance exercise, MuRF-1 *mRNA* expression generally peaks within 1 to 4 h post-exercise, as observed at 1 h [49], 2 h [55], 3 h [36], and 4 h [73]. MuRF-1 *mRNA* expression typically returns to baseline within 24 h [56,73]. The transient activation of MuRF-1 *mRNA* following resistance exercise is driven by mechanical stress that activates specific signaling pathways, such as PI3K-Akt and FoxO, to coordinate translational activity and increase protein synthesis [11,12,74]. This process triggers the release of anabolic hormones that modulate intracellular signaling [4,33]. Exercise stimulates the redistribution of nutrients and energy to skeletal muscle, promoting the activation of pathways such as PI3K-Akt, which negatively regulates FoxO to suppress excessive protein degradation [28,48,74]. Concurrently, mechanical stress activates the ubiquitin–proteasome system (UPS) through the upregulation of MuRF-1, a process necessary to replace damaged proteins, maintain intracellular protein balance, and prevent the accumulation of misfolded proteins during the recovery period [14,68]. The balance between protein synthesis and degradation is controlled by the interaction of FoxO, MuRF-1, and the UPS, ensuring that protein turnover supports hypertrophy without compromising cellular homeostasis [10,11,12,13,74]. These interactions underscore the highly coordinated nature of the adaptive mechanisms in skeletal muscle, reflecting the specific demands imposed by resistance exercise, while endurance exercise induces a variety of metabolic and morphological changes by activating distinct molecular pathways [62,75,76].

Regarding endurance exercise, MuRF-1 *mRNA* expression typically peaks between 40 min and 4 h post-exercise, as observed at 40 min [54], 2 h [49], 2.5 h [68], 3 h [61,62], and 4 h [40]. MuRF-1 *mRNA* expression normalized to baseline levels by 24 h after exercise [49]. Endurance exercise induces a transient upregulation of MuRF-1 *mRNA* expression, primarily mediated by metabolic stress and energy demand signaling pathways such as adenosine monophosphate-activated protein kinase (AMPK) and FoxO [28,74,75]. The activation of AMPK, triggered by an elevated adenosine monophosphate (AMP) and adenosine triphosphate (ATP) ratio during prolonged exercise, acts as a key metabolic sensor that regulates energy homeostasis, facilitating catabolic processes while it suppresses anabolic pathways [11,69]. Simultaneously, FoxO transcription factors are activated under conditions of energy stress, driving the expression of MuRF-1 to facilitate the degradation of damaged or misfolded proteins via the UPS [4,45,74]. This selective proteolytic activity is essential for maintaining proteostasis and adapting the muscle to repetitive mechanical and metabolic demands, which underscores MuRF-1’s pivotal role in protein turnover regulation and supporting endurance-specific muscular adaptations [46,50,76]. Moreover, this molecular regulation ensures that skeletal muscle sustains its structural and functional integrity during extended exercise periods, reduces damage accumulation, and optimizes mitochondrial biogenesis [45,60,74].

In a practical context, endurance exercise performed for an extended period induces significant structural and physiological adaptations at the muscular level [45,68]. These include increased sarcomere length, enhanced perimysial connective tissue, and improved mitochondrial efficiency, thus contributing to enhanced substrate utilization and oxidative capacity [74,77]. These adaptations allow skeletal muscle to tolerate workloads more effectively and reduce muscle protein breakdown during recovery [11,50,78]. Furthermore, moderate endurance exercise has been shown to produce significant alterations in MuRF-1 *mRNA* expression in trained soldiers [62]. MuRF-1 *mRNA* levels increased significantly immediately after exercise (4.7-fold), and there was an effect that persisted 3 h (5.7-fold) after 60 min of cycling at 60 ± 5% VO_2peak_. Harber et al. [40] found that after 45 min of a treadmill run at 75% VO_2max_, MuRF-1 *mRNA* expression was upregulated at 4 h post-exercise. The results of these studies showed that training variables such as intensity and volume may have contributed to the observed gene expression response to endurance exercise. MuRF-1 is a highly homologous protein related to a part of a myocellular structure linked to a mechanosensory function [1,4]. This role is particularly significant in the context of acquired exercise tolerance, as this adaptive process is dependent on exercise intensity [1,3]. Collectively, these mechanisms highlight the pivotal role of MuRF-1 as a molecular regulator of endurance training adaptations, orchestrating metabolic, structural, and molecular responses to support muscle integrity, optimize recovery, and enhance performance under sustained physical demands.

Combined activities integrating resistance and endurance exercises exert complex effects on the regulation of MuRF-1 mRNA, influenced by factors such as exercise order, intensity, and mode [11,68]. The sequence of exercises plays a critical role in molecular responses [31,54]. Studies suggest that when endurance exercise precedes resistance exercise, AMPK is activated, and it competes with the Akt and mammalian target of rapamycin (mTOR) pathway and potentially reduces the anabolic stimulus [28,37]. Conversely, resistance exercise performed before endurance exercise minimizes this interference, preserves Akt/mTOR activation, and limits competition with AMPK, which ultimately enhances the anabolic response [64,74]. In acute studies, Coffey et al. [32] observed a significant increase in MuRF-1 *mRNA* expression 3 h post-exercise following consecutive resistance exercise (8 × 5 leg extensions at 80% 1-RM) and repeated sprints (10 × 6 s maximal effort) performed alternately. Similarly, Hansson et al. [39] and Lundberg et al. [53] demonstrated an acute response to combined endurance (45 min cycling) and resistance exercise (4 × 7 knee extensions), with increased MuRF-1 *mRNA* expression at 3 h in the elbow extensors and lower limbs, respectively. In contrast, Lysenko et al. [54] reported no significant increase when intermittent aerobic cycling (70 min) was followed by one-leg strength exercise (four sets of knee extensions until exhaustion). These data suggest that combining strength exercises immediately after intense aerobic activity may impair subsequent aerobic performance if applied chronically. However, this effect may be mitigated if training variables are planned appropriately [37].

Louis et al. [49] examined the time course of MuRF-1 *mRNA* after an acute bout of resistance and endurance exercise. Both exercises significantly increased MuRF-1 *mRNA* expression compared with the baseline period. In addition, the two exercise protocols showed differences in the peak of MuRF-1 *mRNA* levels, with resistance at 1 h (3.5-fold) and endurance exercise at 2 h (3.6-fold). These MuRF-1 *mRNA* time course results were confirmed in another study that evaluated the protein levels of molecular markers of the ubiquitin–proteasome system [68]. Thus, it was observed that the MuRF-1 *mRNA* levels in single-bout endurance exercise were significantly higher at 0 h, 2.5 h, and 22 h compared with single-bout resistance exercise. Furthermore, single-bout endurance exercise increased MuRF-1 *mRNA* at 2.5 h from basal levels (*p* < 0.001) and between exercise groups (340 ± 94%, *p* < 0.001). Normally, the post-exercise period is the time when protein synthesis and degradation processes interact to promote muscle maintenance or even hypertrophy, as well as to help tissue regeneration and repair, contributing to muscle recovery after exercise [6,10,11]. Lundberg et al. [51] reported modest reductions in MuRF-1 *mRNA* shortly after exercise (15 min and 3 h), suggesting an acute response that may not differ substantially between endurance (AE) + resistance (RE) and RE alone. However, Fernandez-Gonzalo et al. [35] demonstrated that in an untrained state, AE + RE resulted in significantly higher MuRF-1 *mRNA* expression compared with RE alone, indicating that the addition of aerobic exercise might potentiate the acute molecular response in untrained individuals. In contrast, Lundberg et al. [52], evaluating MuRF-1 *mRNA* levels 72 h post-training, found no significant differences between protocols, implying that the acute effects of AE + RE may not translate into prolonged responses. Supporting the role of exercise order, Coffey et al. [31] observed increased MuRF-1 *mRNA* expression when endurance exercise followed resistance exercise, reinforcing that exercise sequencing can modulate molecular responses. Although intracellular stress generated by muscle contractions differs between these two modes, evidence suggests that both may regulate similar gene targets and biological processes [77], which could be further influenced by nutrient interventions [11]. Such conditions may enhance recovery efficiency after exercise by stimulating skeletal muscle protein turnover and net protein gain, a continuous cellular process regulated by the balance between muscle protein synthesis and protein breakdown [10,12].

Skeletal muscle atrophy is associated with muscle protein breakdown [3,4], and physical exercise has been recognized as an effective intervention for maintaining whole-body health and attenuating the loss of lean mass in older adults [46,67,71]. Evidence demonstrates that baseline MuRF-1 *mRNA* expression is significantly higher in older women (85.2 ± 1.7 years) than in younger women (23.4 ± 1.7 years) after resistance exercise [65], suggesting an age-related elevation in proteolytic activity [33,56,72]. This upregulation may reflect an adaptive response aimed at maintaining protein turnover and muscle homeostasis in an environment of anabolic resistance. Similarly increased proteolytic gene expression was verified in older women (85.0 ± 1.6 years), while the basal level of MuRF-1 *mRNA* decreased in younger women (24.0 ± 2.0 years) after 12 weeks of progressive resistance training [72]. In contrast, no effect of exercise on MuRF-1 *mRNA* expression was observed in older (average 80 years) and younger (average 26 years) women after 12 weeks of resistance exercise training [38].

Experimental factors, such as methodological differences in the exercise protocols, may partially explain this divergence in results. In both studies, the participants performed 3 sets ×10 repetitions at 70–75% of the 1-RM, and the muscle biopsies were obtained before and 4 h after resistance exercise [65], while in the other experimental design, they were collected before and immediately after an acute bout of resistance exercise [72]. In another study, participants performed 6 sets ×20 maximal voluntary contractions, with muscle biopsies collected at rest and 2.5 h post-resistance exercise [38]. Interestingly, other studies with older and younger subjects found an upregulation of MuRF-1 *mRNA* expression at 2 h [67], 3 h, and 6 h after resistance exercise [36], showing that, perhaps, specific exercise protocols can effectively promote changes in MuRF-1 *mRNA* levels. Consistently with previous findings, MuRF-1 *mRNA* expression was significantly greater in older men (68.0 ± 1.0 years) than in younger men (21.0 ± 1.0 years) at baseline [33]. According to Merritt et al. [56], MuRF-1 *mRNA* levels were higher in AGE76 (75.5 ± 0.7 years) than in AGE40 (40.4 ± 1.1 years) at baseline. Both results corroborate the findings of other studies [65,72] and suggest that both genders’ display of a similar average age and training status may be their response to a resistance exercise stimulus in a similar manner. Older adults exhibited an attenuated MuRF-1 responses to resistance exercise compared to younger individuals, reflecting reduced proteolytic activity and muscle plasticity [65,67]. This diminished response may be linked to declines in anabolic hormones, which are crucial for Akt and FoxO signaling regulation [72]. Furthermore, the lower muscle mass observed in the elderly correlates with reduced basal MuRF-1 *mRNA* expression, potentially indicating impaired protein degradation demands [46]. Understanding the molecular mechanisms activated by exercise is essential for developing physical activity interventions aimed at mitigating skeletal muscle loss during aging.

This review has several limitations. First, most included studies demonstrated moderate methodological quality, with PEDro scores ranging from 3 to 7, indicating a risk of bias. Second, there was high variability in exercise protocols, including differences in the type (resistance, endurance, or combined), training variables (e.g., intensity), and timing of assessments, which hindered direct comparisons of the findings. Third, the small number of randomized controlled trials (RCTs) reduced the robustness of the evidence base, and the inclusion of specific populations, such as older adults and women, was limited, leaving gaps in the understanding of how variables such as age and sex influence the outcomes. However, this is the first systematic review to exclusively focus on studies involving healthy humans, excluding potential confounding results derived from animal models or individuals with medical conditions. The inclusion of studies encompassing a wide range of exercise protocols enabled a comprehensive exploration of how various variables, such as exercise type, intensity, and recovery time, affect MuRF-1 *mRNA* expression. Furthermore, diverse experimental designs provide insights into the molecular mechanisms underlying proteolytic gene expression.

In summary, this systematic review provides insights into the specificity of MuRF-1 *mRNA* expression in response to different exercise modalities. The findings indicate an early acute skeletal muscle response to resistance and endurance exercise across various fitness levels, with peak expression occurring primarily within 1–4 h post-exercise. Notably, the combination of resistance and endurance exercise has a greater effect on MuRF-1 *mRNA* expression at 3 h post-exercise than resistance exercise alone. Resistance and endurance exercises showed a similar temporal pattern, with MuRF-1 *mRNA* levels returning to baseline within 24 h post-exercise. These observations contribute to the understanding of the molecular basis of adaptive responses elicited by distinct exercise modalities. The central role of MuRF-1 in muscle mass regulation underscores its relevance for clinical practice, rehabilitation, and athletic training. In clinical settings, understanding MuRF-1’s molecular responses can guide the development of personalized strategies for preventing muscular atrophy in older adults, reducing the impact of sarcopenia, preserving functional capacity, and improving quality of life. In sports, insights into MuRF-1 *mRNA* expression changes may inform the optimization of training protocols to minimize muscle protein breakdown and enhance anabolic responses, particularly during periods of intense competition. Key exercise variables, such as intensity, volume, and sequence, play a crucial role in the molecular regulation of MuRF-1, highlighting the importance of tailored approaches by health professionals and coaches to promote adaptations and superior outcomes. Adjusting these variables effectively supports muscle recovery, improves physical performance, and addresses the specific needs of diverse populations. Furthermore, these results emphasize the complexity of molecular responses induced by acute and chronic exercise, underscoring the need for further research to elucidate the regulatory mechanisms within the intracellular signaling pathways responsible for maintaining skeletal muscle mass. Since *mRNA* expression represents just one component of the multifaceted gene regulation processes in skeletal muscle, future studies should explore transcriptional and post-transcriptional mechanisms to provide a more comprehensive understanding of these adaptations.

Finally, future studies should simultaneously measure the effects of MuRF-1 *mRNA* and other mechanisms, such as by evaluating the time course of MuRF-1 *mRNA* through longitudinal designs, genetic polymorphisms, epigenetic factors, nutritional supplementation, and metabolic aspects. These investigations will provide insights into transcriptional responses across different exercise modalities and their potential to achieve clinical and performance benefits.

## 5. Conclusions

This systematic review provides evidence that MuRF-1 *mRNA* expression is responsive to physical exercise in humans, exhibiting distinct modulation patterns influenced by the exercise modality, intensity, and sequence. Furthermore, the effects of resistance and endurance exercises were similar, both in terms of MuRF-1 *mRNA* expression and the return of these values to baseline levels after exercise. Aging seems to influence MuRF-1 regulation, while the level of physical fitness impacts the magnitude of molecular responses. Trained individuals showed more attenuated responses, possibly due to greater adaptive muscle efficiency. Sex-related differences indicated that men exhibited higher levels of MuRF-1, likely due to hormonal and physiological factors. By tailoring exercise interventions to these variables, practitioners can maximize the benefits of physical activity for muscle health, functional performance, and long-term adaptability. These findings offer insights into the relationship between physical exercise and molecular regulation, establishing a foundation for future research to further explore these mechanisms and their applications in clinical and athletic settings.

## Figures and Tables

**Table 1:** Summary of reviewed studies (*n* = 46 studies).

Author	Aim	Participants	Biopsy Time Points	Exercise Protocol	MuRF-1 Main Effects
Apró et al., 2015 [28]	Investigation of whether increased AMPK activity from high-intensity interval cycling suppresses mTORC1 signaling induced by resistance exercise in well-trained individuals	8 healthy trained men (age: 26.0 ± 2.0)	Baseline (pre), 90 and 180 min after exercise	ER (5 sets × 4 min intervals at 85% VO_2max_ on the cycle ergometer), and R (4 sets × 8–10 repetitions at 80% 1-RM, 4 sets × 10–12 repetitions at 70% 1-RM, and 2 sets to volitional fatigue at 60% 1-RM on the leg press machine)	MuRF-1 was unchanged in the R trial, but increased 2.2- and 1.6-fold (*p* < 0.05) at 90 and 180 min following resistance exercise in the ER trial
Baumert et al., 2022 [29]	Analysis of the polygenic association with EIMD, and evaluation of whether SNPs linked to in vivo EIMD were also associated with the repair rate in vitro human skeletal muscle	In vivo: 65 healthy and untrained (age: 22.5 ± 4.0), men (*n* = 26) and women (*n* = 39). In vitro: 12 subjects (*n* = 8 men, *n* = 4 women)	Baseline (pre), immediately after (post), and post 48 h	12 sets × 10 maximal eccentric unilateral knee extensors	MuRF-1 was associated with EIMD intervention and demonstrated an increased expression following acute resistance exercise (*p* < 0.05)
Churchley et al., 2007 [30]	Evaluation of whether pre-exercise muscle glycogen influences the transcription of early-response genes regulating muscle growth	7 strength-trained men (age: 30.0 ± 6.7)	Baseline, immediately after RE, and following the 3 h recovery	8 sets × 5 repetitions at 80% 1-RM for each leg	MuRF-1 was higher (3-fold; *p* < 0.05, ES 0.6) in the Norm than in the Low leg at rest
Coffey et al., 2009 [31]	Analysis of the impact of consecutive resistance and endurance exercise on early molecular responses in skeletal muscle	8 physically active men (age: 22.9 ± 6.3)	Baseline, 15 min after each exercise, and following 3 h recovery	RE (8 sets × 5 repetitions on the leg extension at 80% 1-RM) and EE (30 min cycling, 70% VO_2_ peak)	MuRF-1 increased significantly when RE preceded EE (*p* = 0.009)
Coffey et al., 2009 [32]	Quantification of acute cellular responses in skeletal muscle following successive resistance and sprint training sessions, and assessment of the impact of exercise order	6 trained men (age: 24.7 ± 6.3)	Baseline, 15 min after each exercise bout, and following 3 h recovery	RE (8 sets × 5 repetitions on the leg extension at 80% 1-RM) and SPR (10 sets × 6 s maximal effort sprints cycling)	MuRF-1 was elevated above rest from RE1-SPR2, and SPR1-RE2 (ES > 0.1, *p* < 0.01). MuRF-1 was moderately exacerbated when SPRs were undertaken after RE (RE1-SPR2 vs. SPR1-RE2, ~25%, ES = 0.75)
Dalbo et al., 2011 [33]	Analysis of baseline and 24 h post-exercise *mRNA* expression of atrogin-1 and MuRF-1 in young and old men	22 untrained and healthy men. Younger (*n* = 13, age: 21.0 ± 1.0) and older (*n* = 9, age: 68.0 ± 1.0)	Baseline and 24 h after exercise	3 sets × 10 repetitions at 80% of their 1-RM for smith squats, leg press and leg extension	No between-group age differences 24 h after exercise were revealed for MuRF-1, and no significant within-group change in response to the exercise was revealed (*p* > 0.05)
Drummond et al., 2014 [34]	Comparison markers involved in ubiquitin-mediated and autophagic lysosomal proteolysis among older women	Inactive (*n* = 7, age: 83.0 ± 1.8) and active (*n* = 7, age: 77.3 ± 1.7) older women	After performance tests	Maximum voluntary isometric knee extension test	MuRF-1 were lower in inactive, frail older women compared to in active healthy women (*p* = 0.01)
Fernandez-Gonzalo et al., 2013 [35]	Investigation of acute molecular muscle responses pre- and post-5-week training using either AE + RE or RE alone	10 healthy and physically active men (age: 25.0 ± 4.0)	Baseline (pre) and 3 h post-RE	4 sets × 7 maximal knee extension ergometer (RE), and one-legged cycle 40 min at ± 70% of W_max_ at 60 rpm (AE)	MuRF-1 was higher in AE + RE than in RE at PRE (*p* < 0.005). In the trained state, MuRF-1 decreased from PRE to POST in AE + RE with no change in RE (*p* = 0.003)
Fry et al., 2013 [36]	Characterization of the MPB response to exercise via the autophagosome–lysosomal and UPS pathways in younger and older adults	16 younger (8 men and 8 women, age: 27.0 ± 2.0) and 16 older (8 men and 8 women, age: 70.0 ± 2.0)	Baseline, 3, 6, and 24 h following RE	8 sets × 10 repetitions at 70% of 1-RM in the leg extension machine	Following exercise, there was an increase in expression of MuRF-1 at 3 h and 6 h post-exercise in both groups (*p* < 0.05)
Fyfe et al., 2016 [37]	Comparison of the effects of a single session of concurrent exercise, combining HIIT or MICT cycling, on mTORC1 signaling and *mRNA* expression in human skeletal muscle, versus RE alone	8 physically active men (age: 27.0 ± 4.0)	Immediately before RE, 1 and 3 h after exercise protocol	RE (8 sets × 5 repetitions on the leg press at 80% 1-RM), HIIT cycling (10 sets × 2 min at 120% lactate threshold), and MICT cycling (30 min at 80% lactate threshold)	MuRF-1 increased at RE + 3 h for both MICT + RE (535 ± 464%; ES = 0.33 ± 0.20; *p* = 0.016) and HIIT ± RE (585 ± 684%; ES = 0.52 ± 0.64; *p* = 0.170) compared with RE
Greig et al., 2011 [38]	Comparison of baseline muscle properties and anabolic response between younger and older	25 untrained and healthy women. Older (*n* = 9, range: 76–82 years) and younger (*n* = 16, range: 19–30 years)	Baseline and 2.5 h after RE	20 sets × 6 repetitions isometric maximum voluntary contractions	MuRF-1 did not present significant differences between older and younger women (*p* > 0.05)
Hansson et al., 2019 [39]	Investigation of how a prior bout of AE influences molecular signaling in response to RE of the elbow extensors	11 healthy and physically active men (age: 28.0 ± 5.0)	Baseline (pre), 15 min (post1) and 3 h after (post2)	AE (~45 min at 70% peak workload) and RE (4 sets × 7 maximal repetitions)	MuRF-1 was greater from pre to post2 in AE + RE compared with RE (18- vs. 3.5- and 4- vs. 2-fold, respectively, interaction *p* < 0.05)
Harber et al., 2009 [40]	Investigation of the muscle-specific metabolic response to running in relation to muscle growth	8 aerobically trained men (age: 26.0 ± 2.0)	Baseline, 4 h and 24 h after exercise	45 min treadmill run at ~75% VO_2 max_	MuRF-1 was higher at 4 h in the vastus lateralis only (*p* < 0.05)
Hinkley et al., 2017 [41]	Analysis of the impact of short-term intense endurance training influences cycling performance, and the acute and chronic signaling responses of skeletal muscle stress and stability markers	10 healthy and physically active men and women (age: 25.0 ± 2.0)	Baseline and 3 h after the cycle time trial on days 1 and 12	20 km time trial on a cycle ergometer (70–100% VO_2 max_)	Following training (day 12), the acute exercise-induced transcriptional response of MuRF-1 was reduced compared to day 1 (*p* < 0.05)
Jamart et al., 2012 [42]	Examination of protein markers involved in these processes during ultra-endurance running in humans, evaluation of their coordination with the UPS, and identification of signaling pathways regulating these responses	11 aerobically trained men (age: 42.1 ± 7.8)	2 h before starting and immediately after finishing exercise	24 h treadmill protocol	MuRF-1 increased (71 ± 31%, *p* = 0.023), and MuRF-1 protein level (55 ± 26%, *p* = 0.034)
Kamandulis et al., 2022 [43]	Use of repeated DJs as an eccentric contraction model to examine the impact of extending the interval between DJs from 20 s to 5 min	16 healthy untrained men, DJ-20s (age: 30.9 ± 8.5) and DJ-5 min (age: 30.0 ± 6.0)	Baseline (pre) and 1 h after exercise	50 DJs with either a 20 s (DJ-20 s) or 5 min (DJ-5 min) rest between DJs	No significant differences in the MuRF-1 *mRNA* expression was found over time or between the two protocols
Kern et al., 2010 [44]	Comparison of ISO-K and VIB trainings effects on muscle mass and strength	29 physically active men. ISO-K (age: 22.6 ± 3.9) and VIB (age: 23.1 ± 2.7)	Baseline and after the period of 8 weeks of training	Maximal isometric unilateral leg extension, squat jump test, and 30 m acceleration running test	MuRF-1 did not change pre and post training using VIB and ISO-K protocols (*p* > 0.05)
Kim et al., 2011 [45]	Modulation of signaling pathways linked to cellular stress in skeletal muscle following a 200 km run	8 trained men (age: 44.0 ± 1.0)	2 weeks before and 3 h after race	200 km running race	MuRF-1 increased by 583.0% ± 244.3% (*p* = 0.024)
Konopka et al., 2010 [46]	Assessment of molecular markers linked to muscle hypertrophy after aerobic training in aging skeletal muscle	9 older women (age: 70.0 ± 2.0)	Baseline and after 12 weeks of aerobic exercise training	Cycle ergometer 20–45 min at 60–80% heart rate reserve	MuRF-1 was unaltered by aerobic training
Koskinen et al., 2017 [47]	Examine whether submaximal exhaustive exercise activates stress-sensing proteins in three specific sarcomere regions of the titin molecule	10 healthy and physically active men (age: 26.0 ± 6.0)	Immediately and 3 h after the exercise	10 × drop jumps unilaterally until complete exhaustion	MuRF-1 correlated positively post-exercise (r = 0.73, *p* = 0.03) and negatively with jump height after 3 h (r = −0.75, *p* = 0.01).
Léger et al., 2006 [48]	Assessment of active phosphorylated Akt protein and its downstream targets involved in hypertrophy GSK-3β, mTOR, p70^S6K^, 4E-BP1, and atrophy regulation of Foxo1, Foxo3, atrogin-1, and MuRF-1 in human skeletal muscle	25 healthy and physically active men. Strength group (age: 36.8 ± 5.5) and endurance group (age: 32.8 ± 2.5)	Pre-Tr (1 week before RTP), Post-Tr (48–72 h after last session of the 8 week RTP), and Post-de-Tr (8 weeks after the last session)	LOW group performed 4 sets × 3–5 repetitions. HIGH group performed 2 sets × 20–28 repetitions. The exercises were performed in the fixed order (leg press, squat, and leg extension)	Following 8 weeks of RTP, there was a 2.5-fold increase in MuRF-1 in Post-Tr (*p* < 0.01)
Louis et al., 2007 [49]	Time course analysis of proteolytic *mRNA* induction following an acute session of RE or RUN exercise	RE group (2 women and 4 men, age: 25.0 ± 4.0) and RUN group (1 woman and 5 men, age: 25.0 ± 4.0)	Baseline, immediately after protocol, and 1, 2, 4, 8, 12, and 24 h post-exercise	3 sets × 10 repetitions at 70% 1-RM and 30 min of treadmill running at 75% of maximum O_2_ uptake	RE increased (*p* < 0.05) *mRNA* expression of MuRF-1 early (3.5-fold, 1–4 h post-exercise). RUN also increased (*p* < 0.05) MuRF-1 levels (3.6-fold, 1–4 h post-exercise)
Luden et al., 2010 [50]	Evaluation of the physiological impact of a 3-week taper in competitive distance runners	7 trained men (age: 20.0 ± 1.0)	Baseline and after a 3-week taper	8 km cross-country	MuRF-1 increased following exercise before and after taper (*p* < 0.05)
Lundberg et al., 2012 [51]	Impact of an acute aerobic exercise session on molecular responses to subsequent RE	9 healthy and physically active men (age: 23.0 ± 2.0)	Baseline (pre), 15 min (post1) and 3 h after RE (post2)	45 min one-legged cycling at 70% W_max_ 60 rpm (AE), and 4 sets × 7 maximal knee extension (RE)	MuRF-1 showed modest decrease over time (time effect F = 4.0, *p* = 0.038)
Lundberg et al., 2013 [52]	Evaluate if chronic AE + RE induces greater muscle hypertrophy compared to RE alone	10 healthy and physically active men (age: 25.0 ± 4.0)	Baseline and 72 h after training	45 min one-legged cycling at 70% W_max_ 60 rpm (AE), and 4 sets × 7 maximal knee extension (RE)	MuRF-1 did not present differences between pre- and post-exercise (*p* > 0.05)
Lundberg et al., 2014 [53]	Examination of acute and chronic effects of consecutive AE + RE sessions compared to RE alone	10 healthy and physically active men (age: 26.0 ± 5.0)	Baseline (pre) and 3 h after (post)	45 min one-legged cycling at 70% W_max_ 60 rpm (AE), and 4 sets × 7 maximal knee extension (RE)	MuRF-1 increased after AE + RE (2.9-fold, *p* = 0.003), while remaining stable after RE alone (interaction: F = 20.4, *p* = 0.001)
Lysenko et al., 2016 [54]	Assessment of whether strength exercise following intermittent aerobic exercise activates pathways regulating mitochondrial biogenesis, protein synthesis, and proteolysis in trained skeletal muscle	9 amateur endurance-trained (age: 18 to 30)	Baseline, 40 min, 5 and 22 h after the aerobic exercise	Cycling (~45 min, 60–95% AT) and strength exercise with one-leg extension at the knee joint (4 sets × 10–12 repetitions at 75% 1-RM), while the other leg was resting	MuRF-1 was only increased after the aerobic exercise (40 min, 2.4-fold, *p* = 0.05), and remained the same after combined load
Mascher et al., 2008 [55]	Assessment of whether two training sessions separated by 48 h differentially impact pathways in these opposing processes	8 healthy men (age: 23.0 ± 1.0)	Baseline, 15 min, 1 h, and 2 h after exercise	4 sets × 10 repetitions at 80% of 1-RM in the leg press machine	MuRF-1 increased after exercise, 30% lower after the second exercise session than after the first one (*p* < 0.05)
Merritt et al., 2013 [56]	Evaluation of skeletal muscle proinflammatory signaling at rest and 24 h after unaccustomed knee extension contractions causing muscle damage	87 subjects: AGE40 (*n* = 38, 19 women and 19 men, age: 40.4 ± 1.1), AGE61 (*n* = 27, 18 women and 9 men, age: 61.2 ± 0.6), and AGE76 (*n* = 22, 15 women and 7 men, age: 75.5 ± 0.7)	Baseline and 24 h after RE	9 sets × 10 repetitions of bilateral knee extensions against a resistance load equal to 40% MVC, 65% of 1-RM	MuRF-1 was higher in AGE76 compared with AGE40 at baseline (*p* < 0.05), and both age groups decreased in MuRF-1 to similar levels 24 h after unaccustomed RE (*p* < 0.05)
Michel et al., 2023 [57]	Identification of mechanisms linked to phenotypic responses on skeletal muscle proteolytic markers	11 untrained men (age: 18 to 30)	Baseline (pre), after 7 weeks of RT (MID), and after 7 weeks of HIIT (post)	(RT) were 6–10 sets ×6 repetitions (70–95% 1-RM), and (HIIT) were 5−10 sets × 1 min running at a high intensity	MuRF-1 showed model significance (*p* = 0.002), with post levels exceeding both pre (*p* = 0.004) and MID (*p* = 0.032)
Mikkelsen et al., 2017 [58]	Examination of whether intramuscular inflammatory and anabolic/catabolic signaling correlates with age- and training-related changes in muscle composition, including the distribution of contractile and non-contractile tissue	49 untrained men, 12 Y-Un (24.0 ± 3.0) and 12 O-Un (66.0 ± 4.0). Trained individuals, 10 Y-Tr (26.0 ± 4.0) and 15 O-Tr (64.0 ± 4.0)	After exercise protocol	Knee extensor muscle strength was performed on the non-dominant leg	MuRF-1 displayed a significant age × training interaction (*p* = 0.022), with a lower expression in O-Tr compared to both Y-Tr (*p* = 0.013) and O-Un (*p* < 0.001)
Moberg et al., 2021 [59]	Analysis of acute molecular responses to concurrent exercise targeting different muscles	8 healthy trained men (age: 31.5 ± 5.0)	Baseline, immediately, 90 and 180 min following exercise	EE: 5 × 4 min intervals at 83 ± 3% of VO_2peak_. RE: 10 sets × 9–12 repetitions until final fatigue 10-RM	MuRF-1 at 90 and 180 min were higher than baseline (*p* < 0.05)
Murach et al., 2014 [60]	Analysis of gene expression changes in gastrocnemius MHC I and MHC IIa muscle fibers during two distinct training phases	6 trained men (age: 20.0 ± 1.0)	4 h post 8 km run (heavily trained and tapered)	8 km cross-country	MuRF-1 unchanged during exercise in the heavily trained and tapered states
Nedergaard et al., 2007 [61]	Examination of protein degradation by analyzing the expression of UPS components following repeated exercise bouts	20 healthy men (age: 23.8 ± 2.8). Step (*n* = 7), step + weight (*n* = 7) and control (*n* = 6) groups	1 week before each bout, 3 h after, 24 h after, and 7 days post-exercise	30 min of bench stepping, performing eccentric work with one and concentric work with the other leg	MuRF-1 showed strong upregulation after 3 h (*p* < 0.001)
Pasiakos et al., 2010 [62]	Characterization of the molecular response related to skeletal muscle growth and atrophy following a single session of moderate endurance exercise in adult men	10 trained men (age: 23.0 ± 1.0)	Immediately (0 h) and 3 h after exercise	60 min of upright cycling at 60 ± 5% VO_2 peak_	MuRF-1 increased 4.7- and 5.7-fold 0 h and 3 h post-exercise, respectively, compared with the resting time points (*p* < 0.001)
Popov et al., 2018 [63]	Assessment of the impact of a 2-month aerobic training program on baseline parameters in human muscle	10 untrained men (age: 21–26)	Baseline (pre) and after the 2-month training program, 1 and 4 h after the one-legged knee extension exercise	One-legged continuous knee extension exercise (55 min at 75% AT)	MuRF-1 in the endurance-trained state was lower than in the untrained state (*p* < 0.01)
Pugh et al., 2015 [64]	Investigation of how an acute HIIT session influences molecular responses to resistance exercise in untrained skeletal muscle	10 healthy and untrained men (age: 21.3 ± 1.0)	Baseline, 2 and 6 h post-RE	RE (4 sets × 8 repetitions on the leg extension at 70% 1-RM), and RE + HIIT (10 sets × 1 min at 90% HR_max_)	MuRF-1 was higher in RE + HIIT compared to RE at both 2 and 6 h (*p* < 0.05)
Raue et al., 2007 [65]	Analysis of *mRNA* expression of proteolytic genes at rest in young and older women, and evaluation of their response to an acute RE session	A group of healthy OW (*n* = 6, age: 85.2 ± 1.3) and YW (*n* = 8, age: 23.4 ± 1.7)	Baseline and 4 h after RE	3 sets × 10 knee extensions at 70% of 1-RM	At rest, MuRF-1 was higher in OW compared to YW (*p* = 0.04). Following RE, both groups showed increased MuRF-1 (*p* = 0.001)
Skelly et al., 2017 [66]	Investigation of sex-based differences in the acute skeletal muscle response to SIT in men and women	10 healthy men (age: 22.0 ± 3.0) and 9 women (age: 22.0 ± 3.0)	Baseline, immediately following exercise, and 3 h after execise	SIT (3 × 20 s cycling efforts)	MuRF-1 increased at 3 h compared to baseline and post-exercise (*p* < 0.001)
Stefanetti et al., 2014 [67]	Analysis of UPS-related gene and protein expression involved in MPB at baseline and 2 h post-RE in older versus younger	10 younger (age: 24.2 ± 0.9) and 10 older (age: 66.6 ± 1.1) healthy and physically active men	2 h after subjects rest (pre-exercise) and 2 h after exercise protocol	3 sets × 14 repetitions at 60% of 1-RM	MuRF-1 was upregulated in both the younger, 1.5-fold, and older, 1.3-fold, groups 2 h following RE (*p* < 0.05) with significant exercise effect (*p* < 0.01)
Stefanetti et al., 2015 [68]	Examination of *mRNA* and/or protein levels of molecular markers of the UPS	18 healthy and untrained men (age: 23.3 ± 0.6). The subjects were divided into ET or RT groups (*n* = 9 per group)	Pre-exercise, 2.5, 5, and 22 h post-exercise	120 min of bicycle exercise at ~60% VO_2 max_ (EE group); 4 sets × 12 repetition 1-RM of three thigh muscle exercises (RE group)	After training, MuRF-1 increased following ET only (*p* < 0.01). In the trained state, single-bout EE increased MuRF-1 at 2.5 h post-exercise (*p* < 0.001)
Valladares-Ide et al., 2019 [69]	Analysis of skeletal muscle signaling activation, protein synthesis, and gene expression of regeneration and degradation markers after repeated eccentric cycling sessions	9 healthy and physically active men (age: 25.4 ± 1.9)	Baseline and 2 h after each bout	2 × 30 cycling bouts at 85% of maximal workload	No changes in MuRF-1 were observed with any cycling exercise
Vann et al., 2021 [70]	Assessment of how AR and PR paradigms impact body composition, serum markers, muscle fiber cross-sectional area, and protein and *mRNA* expression in skeletal muscle	30 trained men, AR (*n* = 16, age: 24.0 ± 2.0) and PR (*n* = 14, age: 24.0 ± 2.0)	Baseline (pre), after 6 weeks RT (post) and after 1 week recovery (DL)	RT protocol (10–32 sets ×10 repetitions at 60% 1-RM). AR group (1–2 sets ×10 repetitions at 60% 1-RM) and PR group (deload)	MuRF-1 was higher at DL compared to pre and post (*p* < 0.001), with post levels also exceeding pre levels (*p* < 0.001)
Whitman et al., 2005 [71]	Investigation of the role of the ubiquitin–proteasome pathway and apoptosis in skeletal muscle wasting in older adults compared to young controls	21 older adults (men = 11, women = 10, age: 72.76 ± 8.31) and 21 young controls (men = 10, women = 11, age: 21.48 ± 2.93)	Post-exercise	Maximal isometric strength at a knee angle of 60°, and isokinetic measurements at velocities of 60, 180, and 300°/s with 4 maximal repetitions with 90 s rest between each testing velocity	MuRF-1 expression remained unchanged across all subject groups
Williamson et al., 2010 [72]	Analysis of Akt–FOXO3A signaling pathway activation before and after a 12-week high-intensity PRT program in young and old women	12 healthy women. YW (*n* = 6, age: 24.0 ± 2.0) and OW (*n* = 6, age: 85.0 ± 1.0)	Baseline and immediately after RE	PRT and exercise bout were 3 sets ×10 repetitions at 70–75% 1-RM	MuRF-1 was downregulated in young women (−29%) following the PRT (*p* < 0.05)
Yang et al., 2006 [73]	Examine *mRNA* expression changes in proteolytic markers in human slow- and fast-twitch muscle fibers after an RE session	8 young healthy and untrained men (age: 23.0 ± 2.0)	Baseline (pre) RE, 4 and 24 h after RE	3 sets ×10 repetitions of bilateral knee extensions at 65% of 1-RM	MuRF-1 increased 4 h post-RE in both MHC I (2.2-fold) and IIa fibers (4.8-fold) (*p* < 0.05)

AE (aerobic exercise), AR (active recovery), AT (anaerobic threshold), EE (endurance exercise), ER (interval cycling followed by resistance exercise), ER-Arm (resistance exercise), ET (endurance training), EIMD (exercise-induced muscle damage), DJs (drop jumps), FOXO1 and FOXO3 (forkhead box O family transcription factors), GSK-3β (glycogen synthase kinase-3β), h (hours), HIIT (high-intensity interval training), ISO-K training (isokinetic exercise), km (kilometers), MHC I (slow-twitch myosin heavy chain), MHC IIa (fast-twitch myosin heavy chain), MICT (moderate-intensity continuous training), min (minutes), *mRNA* (messenger RNA), mTOR (mammalian target of rapamycin), p70^S6K^ and 4E-BP1 (anabolic targets), MuRF-1 (muscle-specific RING finger protein 1), MPB (muscle protein breakdown), OW (old women), O-Un (old untrained), O-Tr (old trained), Post-TR (post training), Post-de-TR (post de-training), Pre-TR (pre training), PR (passive recovery), PRT (progressive resistance training), R (resistance exercise only), R-Arm (resistance exercise only), RE (resistance exercise), RT (resistance training), RM (repetition maximum), RTP (resistance training program), RUN (submaximal running), SIT (sprint interval training), SPR (repeated sprints), UPS (ubiquitin-proteasome system), VIB training (vibrational-proprioceptive stimulation), VO2 max (maximum oxygen volume), Wmax (maximal workload), Y-Un (young untrained), Y-Tr (young trained), YW (young women).

**Table 2:** Risk of bias assessment—PEDro scale (*n* = 46).

Study	A	B	C	D	E	F	G	H	I	J	Score (0–10)
Apró et al., 2015 [28]	Y	N	Y	N	N	N	Y	N	Y	Y	5
Baumert et al., 2022 [29]	N	N	Y	N	N	N	Y	N	Y	Y	4
Churchley et al., 2007 [30]	N	N	Y	N	N	N	Y	N	Y	Y	4
Coffey et al., 2009 [31]	Y	N	Y	N	N	N	Y	N	Y	Y	5
Coffey et al., 2009 [32]	Y	N	Y	N	N	N	Y	N	Y	Y	5
Dalbo et al., 2011 [33]	N	N	N	N	N	N	Y	N	Y	Y	3
Drummond et al., 2014 [34]	N	N	Y	N	N	N	Y	N	Y	Y	4
Fernandez-Gonzalo et al., 2013 [35]	N	N	N	N	N	N	Y	N	Y	Y	3
Fry et al., 2013 [36]	N	N	N	N	N	N	Y	N	Y	Y	3
Fyfe et al., 2016 [37]	Y	N	Y	N	N	N	Y	N	Y	Y	5
Greig et al., 2011 [38]	N	N	N	N	N	N	Y	N	Y	Y	3
Hansson et al., 2019 [39]	Y	N	Y	N	N	N	Y	N	Y	Y	5
Harber et al., 2009 [40]	N	N	N	N	N	N	Y	N	Y	Y	3
Hinkley et al., 2017 [41]	N	N	N	N	N	N	Y	N	Y	Y	3
Jamart et al., 2012 [42]	N	N	N	N	N	N	Y	N	Y	Y	3
Kamandulis et al., 2022 [43]	Y	N	Y	N	N	N	Y	N	Y	Y	5
Kern et al., 2010 [44]	Y	N	Y	N	N	N	Y	N	Y	Y	5
Kim et al., 2011 [45]	N	N	N	N	N	N	Y	N	Y	Y	3
Konopka et al., 2010 [46]	N	N	N	N	N	Y	Y	N	Y	Y	4
Koskinen et al., 2017 [47]	N	N	N	N	N	N	Y	N	Y	Y	3
Léger et al., 2006 [48]	N	N	Y	N	N	N	Y	N	Y	Y	4
Louis et al., 2007 [49]	N	N	Y	N	N	N	Y	N	Y	Y	4
Luden et al., 2010 [50]	N	N	N	N	N	N	Y	N	N	N	3
Lundberg et al., 2012 [51]	Y	N	Y	N	N	N	Y	N	Y	Y	5
Lundberg et al., 2013 [52]	Y	N	Y	Y	N	Y	Y	N	Y	Y	7
Lundberg et al., 2014 [53]	Y	N	Y	Y	N	Y	Y	N	Y	Y	7
Lysenko et al., 2016 [54]	N	N	Y	N	N	N	Y	N	Y	Y	4
Mascher et al., 2008 [55]	N	N	Y	N	N	N	Y	N	Y	Y	4
Merritt et al., 2013 [56]	N	N	Y	N	N	Y	Y	N	Y	Y	5
Michel et al., 2023 [57]	N	N	Y	N	N	Y	Y	N	Y	Y	5
Mikkelsen et al., 2017 [58]	N	N	Y	N	N	Y	Y	N	Y	Y	5
Moberg et al., 2021 [59]	Y	N	Y	N	N	N	Y	N	Y	Y	5
Murach et al., 2014 [60]	N	N	N	N	N	N	Y	N	Y	Y	3
Nedergaard et al., 2007 [61]	N	N	N	N	N	N	Y	N	Y	Y	3
Pasiakos et al., 2010 [62]	N	N	Y	N	N	N	Y	N	Y	Y	4
Popov et al., 2018 [63]	N	N	Y	N	N	N	Y	N	Y	Y	4
Pugh et al., 2015 [64]	Y	N	Y	N	N	N	Y	N	Y	Y	5
Raue et al., 2007 [65]	N	N	Y	N	N	N	Y	N	Y	Y	4
Skelly et al., 2017 [66]	N	N	Y	N	N	N	Y	N	Y	Y	4
Stefanetti et al., 2014 [67]	N	N	Y	N	N	N	Y	N	Y	Y	4
Stefanetti et al., 2015 [68]	Y	N	Y	N	N	N	Y	N	Y	Y	5
Valladares-Ide et al., 2021 [69]	N	N	N	N	N	N	Y	N	Y	Y	3
Vann et al., 2021 [70]	Y	N	Y	N	N	Y	Y	N	Y	Y	6
Whitman et al., 2005 [71]	N	N	N	N	N	N	Y	N	Y	Y	3
Williamson et al., 2010 [72]	N	N	Y	N	N	N	Y	N	Y	Y	4
Yang et al., 2006 [73]	Y	N	Y	N	N	N	Y	N	Y	Y	5

Y = yes; N = no; A = random allocation; B = concealed allocation; C = baseline comparability; D = blind subject; E = blind therapist; F = blind assessor; G = adequate follow-up; H = intention-to-treat analysis; I = between-group comparisons; J = point estimates and variability.

## Data Availability

All data used in this study are included in the manuscript.

## Supplementary Materials

The following supporting information can be downloaded at https://www.mdpi.com/article/10.3390/genes16020153/s1: Table S1: The PRISMA 2020 statement: An updated guideline for reporting systematic reviews; Table S2: Example of search strategy.
