# Supplementary material for: Effects of Physical Exercise on MuRF-1/TRIM63 mRNA Expression in Humans: A Systematic Review
Source: Genes (Basel). 2025 Jan 26;16(2):153. doi: 10.3390/genes16020153 (PMC11855204; doi:10.3390/genes16020153)
Supplement: Supplementary file 1 [file genes-16-00153-s001.zip › genes-3412773-Tables.pdf]

Supplementary Material

Effect of physical exercise on MuRF-1/TRIM63 mRNA expression in humans: A systematic review

Leonardo Henrique Silva Fagundes [1,*], Eduardo Mendonça Pimenta [1], Varley Teoldo da Costa [1]

[1] Sport Psychology Laboratory, UFMG Soccer Science Center, Department of Sport Sciences, Universidade Federal de Minas Gerais (UFMG), 31.270-901, Belo Horizonte, Brazil; leohsf31@hotmail.com; empimenta@uol.com.br; vtcosta@hotmail.com

* Correspondence: leohsf31@hotmail.com

Supplementary Tables

1 Table S1. PRISMA 2020 Checklist

Please see the separate Word document named " PRISMA 2020 Checklist ".

2 Table S2. Example Search Strategy

Database	Search strategy	Results
**Pubmed**	#1: ("muscle ring finger protein 1" [MeSH Terms] OR "MuRF-1" [Title/Abstract] OR "TRIM63" [Title/Abstract] OR "atrogenes" [Title/Abstract] OR "proteolytic gene expression" [Title/Abstract])<br><br>#2: ("physical exercise" [MeSH Terms] OR "endurance training" [Title/Abstract] OR "endurance exercise" [Title/Abstract] OR "resistance training" [Title/Abstract] OR "resistance exercise" [Title/Abstract])<br><br>#3: ("humans" [MeSH Terms] OR "human skeletal muscle" [Title/Abstract] OR "men" [Title/Abstract] OR "women" [Title/Abstract])<br><br>#1 AND #2 AND #3	43
**Scopus**	("muscle ring finger protein 1" OR "MuRF-1" OR "TRIM63" OR "atrogenes" OR "proteolytic gene expression") AND ("physical exercise" OR "endurance training" OR "endurance exercise" OR "resistance training" OR "resistance exercise") AND ("humans" OR "human skeletal muscle" OR "men" OR "women")	59

**Cochrane Library**	("muscle ring finger protein 1" OR "MuRF-1" OR "TRIM63" OR "atrogenes" OR "proteolytic gene expression") AND ("physical exercise" OR "endurance training" OR "endurance exercise" OR "resistance training" OR "resistance exercise") AND ("humans" OR "human skeletal muscle" OR "men" OR "women")	20
**Google Scholar**	("MuRF-1" OR "TRIM63") AND ("physical exercise") AND ("human skeletal muscle")	863
**Web of Science**	("muscle ring finger protein 1" OR "MuRF-1" OR "TRIM63" OR "atrogenes" OR "proteolytic gene expression") AND ("physical exercise" OR "endurance training" OR "endurance exercise" OR "resistance training" OR "resistance exercise") AND ("humans" OR "human skeletal muscle" OR "men" OR "women")	156

* All searches were carried out on December 1, 2024.
